# Standardization of Chemically Selective Atomic Force Microscopy for Metal Oxide Surfaces

**DOI:** 10.1021/acsnano.4c03155

**Published:** 2024-08-05

**Authors:** Philipp Wiesener, Stefan Förster, Milena Merkel, Bertram Schulze Lammers, Harald Fuchs, Saeed Amirjalayer, Harry Mönig

**Affiliations:** † Universität Münster, Physikalisches Institut, Münster 48149, Germany; ‡ Center for Nanotechnology, Münster 48149, Germany; § Martin-Luther-Universität Halle-Wittenberg Institut für Physik, Halle 06120, Germany; ∥ Universität Münster, Physikalisches Institut, Münster 48149, Germany; ⊥ Center for Multiscale Theory and Computation, Münster 48149, Germany

**Keywords:** chemical imaging, metal oxide surfaces, defect characterization, noncontact atomic force microscopy (nc-AFM), probe-tip functionalization, oxygen-terminated copper tip (CuOx-tip)

## Abstract

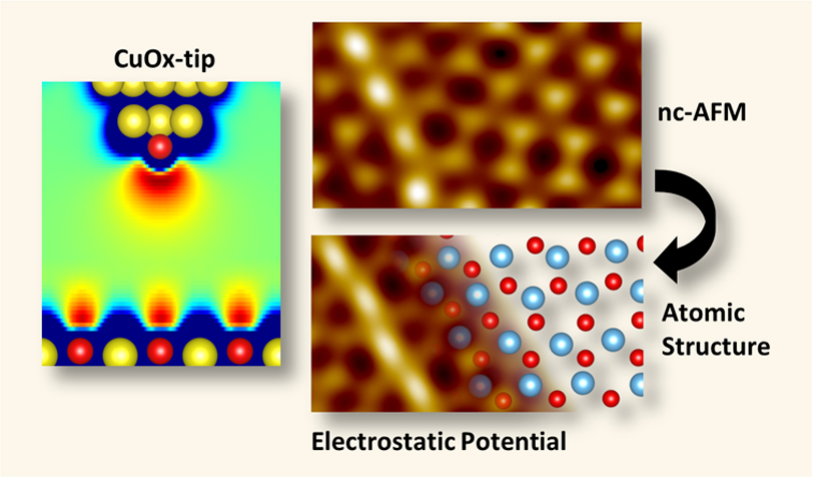

The structures of metal oxide surfaces and inherent defects are vital for a variety of applications in materials science and chemistry. While scanning probe microscopy can reveal atomic-scale details, elemental discrimination usually requires indirect assumptions and extensive theoretical modeling. Here, atomic force microscopy with O-terminated copper tips on a variety of sample systems demonstrates not only a clear and universal chemical contrast but also immediate access to the atomic configuration of defects. The chemically selective contrast is explained by purely electrostatic interactions between the negatively charged tip-apex and the strongly varying electrostatic potential of metal and oxygen sites. These results offer a standardized methodology for the direct characterization of even the most complex metal oxide surfaces, providing fundamental insight into atomic-scale processes in these material systems.

The local atomic structure of metal oxide surfaces largely determines their properties, which are important for a variety of applications ranging from energy conversion and electronics^1−3^ to data storage,^4^ heterogeneous catalysis,^5,6^ and biomedical sciences.^7^ Enabling real-space investigations on the atomic level, scanning probe microscopy methods have become key tools in modern material characterization.^8,9^ In particular, scanning tunneling microscopy (STM) and noncontact atomic force microscopy (nc-AFM) have provided groundbreaking results with regard to surface processes on metal oxides.^10−16^ Yet, the surface structure determination of such compounds remains a considerable obstacle. Even for relatively simple bulk structures, the heterogeneity can lead to complex surface reconstructions and a high degree of disorder, which makes structural elucidation extremely challenging or even intractable.^17^ At the same time, elemental discrimination based on contrast analysis of scanning probe microscopy images can be extremely difficult. Especially the often unknown identity of the probe tip decisively determines the image contrast and limits the reproducibility of such experiments.^18−22^ As a consequence, elemental recognition on the atomic scale usually relies on indirect structural considerations, which are prone to errors and require extensive theoretical modeling of experimental contrasts. This in turn requires assumptions not only about the surfaces themselves but also about the nature of the probe tip.^14,18,19,23,24^

Strongly related to the topography and chemical forces between the tip and the surface, nc-AFM has shown to be a promising tool toward imaging with chemical selectivity. In this regard, Sugimoto et al. investigated a Si(111) surface alloyed with Pb and Sn by force spectroscopy combined with density functional theory (DFT) simulations to discern the heteroatoms on this surface.^25,26^ Taking advantage of the drastically increased resolution by using CO-functionalized tips in conjunction with a qPlus force sensor,^27−31^ Schulz et al. used a combination of nc-AFM, Kelvin-probe AFM, and DFT-based simulations to elucidate a chemical contrast on hexagonal boron nitride.^20^ Liebig et al. investigated a site-selective contrast of CO tips on an ionic CaF_2_(111) surface but also demonstrated imaging artifacts due to dynamic CO-bending effects at reduced tip–sample distances.^21^ In fact, the weak coupling of the CO to the metallic tip base remains a considerable issue.^32−35^ As a complementary approach of nc-AFM tip functionalization, we introduced oxygen-terminated copper tips (CuOx-tips).^36^ Due to the covalent tetrahedral bonding configuration of the terminal oxygen, CuOx-tips are highly rigid, allowing significantly reduction of such artifacts. Furthermore, a systematic comparison of various atomically defined tip terminations (pure metal-, CO-, Xe-, and CuOx-tips) on a partially oxidized Cu(110) (2 × 1)O surface indicated also a chemical-selective (Cu/O)-contrast only for the CuOx-tips. Contrary, CO-tips lead to tip-bending artifacts and suppress the chemical contrast due to their strong passivation as supported by DFT simulations.^22^

In the present work we generalize the outstanding ability of CuOx-tips for chemical imaging of metal oxide surfaces by investigating copper-, silver-, iron-, and titanium oxide systems with a stepwise increasing structural complexity. For all the specific surfaces, metal and oxygen sites and sublattices can be clearly identified in the constant height nc-AFM images and force spectroscopy data. Comparing the nc-AFM contrast from well-known surface structures at different tip heights with the corresponding calculated local electrostatic potentials reveals that the chemically selective contrast is largely dominated by electrostatic tip–sample interactions. Furthermore, we focus on defect structures and surfaces, where so far no conclusive models exist. These results demonstrate that the chemical-selective contrast is universal for metal oxide surfaces, which must be set into context to the longstanding dream in scanning probe microscopy to achieve direct chemical imaging on the single atom level.^20,25,37,38^

## Results and Discussion

**Figure 1:**
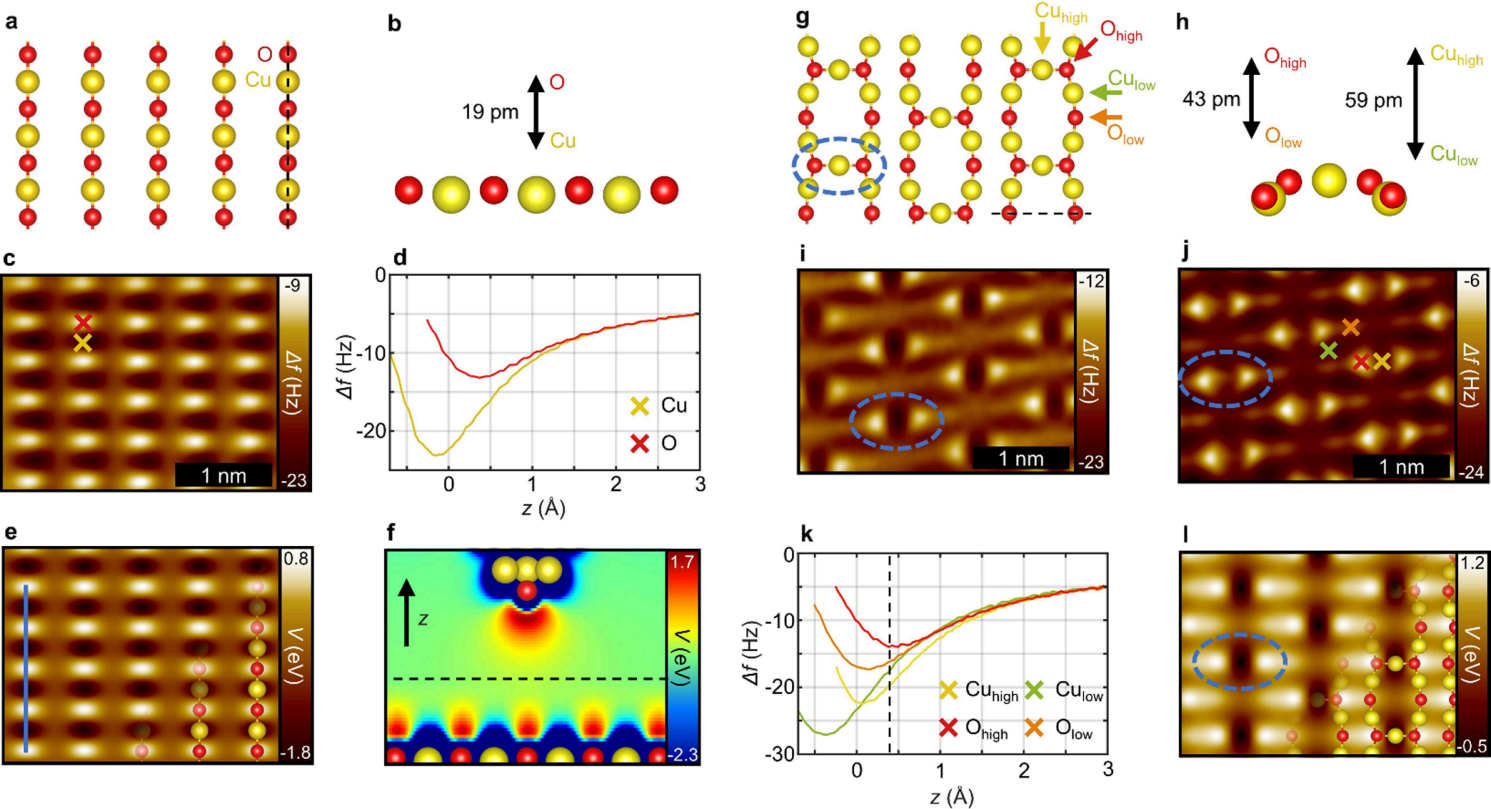
(2 × 1)O reconstruction and (6 × 2)O reconstruction on Cu(110). (a) DFT optimized structure of the (2 × 1)O reconstruction as top view. For clarity, only the top atomic layer is shown. (b) Side view along the dashed line in (a), depicting the relative heights of the surface species. (c) CuOx-tip constant height nc-AFM of the (2 × 1)O reconstruction. (d) Δ*f*(*z*) spectra of the Cu and O sites as marked in (c). *z* = 0 corresponds to the tip height of the measurement in (c). (e) Calculated electrostatic potential of the (2 × 1)O reconstruction, plotted at a height of 162 pm relative to the topmost surface atom. (f) Cross section of the calculated electrostatic potential of the CuOx-tip and the (2 × 1)O surface. As indicated by the dashed line, the data for the tip and surface are obtained from separate DFT calculations. (g) DFT-optimized structure of the (6 × 2)O reconstruction as a top view. (h) Side view along the dashed line in (g), depicting the relative heights of the surface species. (i) CuOx-tip constant height nc-AFM of the (6 × 2)O reconstruction ((j) with reduced tip height). (k) Δ*f*(*z*) spectra of the Cu and O sites marked in (j). *z* = 0 corresponds to the tip height of the measurement in (j), while the dashed line marks the tip height of the measurement in (i). (l) Calculated electrostatic potential of the (6 × 2)O reconstruction, plotted at a height of 162 pm relative to the topmost surface atom.

One of the most basic metal oxide surfaces is the (2 × 1)O reconstruction on Cu(110), which consists of rows of alternating copper and oxygen atoms as depicted by a DFT-optimized structure in Figure 1a,b. A typical CuOx-tip nc-AFM measurement recorded in constant height mode is shown in Figure 1c. It clearly shows the O atoms as bright (repulsive) protrusions and the Cu atoms as dark (attractive) depressions.^22^ This chemical selectivity is further emphasized by Δ*f*(*z*) spectra recorded on the O and Cu sites (Figure 1d), where a distinct separation of the curves over an extended height range can be observed. Specifically, the strongest chemical contrast is obtained at tip height *z* around the Δ*f*(*z*) minimum on the Cu site, where the difference between both Δ*f*(*z*) curves is maximized. To further understand the contrast mechanism, Figure 1e shows the calculated electrostatic potential based on the optimized structure in Figure 1a,b and extracted at a height of about 160 pm above the top-lying surface oxygen (please note that these calculations do not include the tip; for further information, see also the computational details in Methods). The good agreement with the experimentally obtained contrast (Figure 1c) indicates that the chemical selectivity mainly relies on electrostatic interactions between the negative charge of the tip-terminating O atom and the strongly varying charge density of metal and oxygen sites on the surface. Therefore, in this regime of tip–sample distances the metal atoms are dominated by attraction, while the oxygen atoms show a more repulsive interaction with the CuOx-tip. This is also emphasized by a cross section of the electrostatic potential within the tip–sample junction (Figure 1f) and further supported by a comparison of corresponding data for varying tip heights (Figures S1 and S2). For an estimation of the absolute tip–sample distance, we consider the overlap of both the surface and the tip-terminating O atoms charge accumulations toward the tip–sample junction (Figure 1f). Assuming a similar distance for the charge accumulation of the tip of about 160 pm, a rough estimation results in an interaction distance of 320 ± 20 pm between the oxygen atoms of the tip and surface, which is in good agreement with the DFT-derived force–distance curves by Schulze Lammers et al.^22^ (see further discussion in the Supporting Information).

To elucidate how different topographies of oxygen and copper atoms affect the findings from above and to stepwise increase the structural complexity we investigate the well-known (6 × 2)O reconstruction on Cu(110).^39^ It features various metal and oxygen atoms at different relative heights (Figure 1g,h) and allows assessment of how the chemical-selective contrast is affected by topography. Similar to the (2 × 1)O reconstruction, the (6 × 2)O surface comprises rows of alternating copper (Cu_low_) and oxygen atoms (O_low_). In contrast, however, the higher degree of oxidation leads to additional topographically higher copper atoms (Cu_high_) located between these rows, elevating the neighboring oxygen atoms (O_high_).^39^ The resulting height differences are depicted in the side view of the DFT-optimized structure in Figure 1h. At greater tip–sample distances, the nc-AFM measurement (Figure 1i) is dominated by the topographically elevated Cu_high_ and O_high_ sites (blue circle). Again, a site-selective contrast arises for the attractive Cu_high_ atoms and their neighboring, repulsive O_high_ atoms. By approaching further to the surface (Figure 1j), a similar chemical-selective contrast emerges for the underlying rows of Cu_low_ and O_low_ atoms, while the elevated Cu_high_ atoms appear more repulsive due to increasing Pauli repulsion. The corresponding Δ*f*(*z*) spectra of each surface species in Figure 1k highlight the height-dependent evolution of the contrast. Despite the distinct height differences, a clear separation of the Δ*f*(*z*) spectra for the metal and oxygen sites is revealed. Importantly, even the topographically higher Cu_high_ atom remains distinctly more attractive than the topographically lower oxygen sites, indicating robust height sensitivity of the chemical-selective contrast. Figure 1l shows the electrostatic potential for the (6 × 2)O reconstruction, which is dominated by the topographically higher Cu_high_ atoms. Due to their reduced electron density, they appear as dark spots, whereas both oxygen sites appear bright due to an increased electron density, which agrees well with the nc-AFM contrast. In the following, the apparent close relation between CuOx-tip nc-AFM measurements and the electrostatic potential is further investigated by focusing on the R45°O and c(2 × 2)N reconstructions on Cu(100).

**Figure 2:**
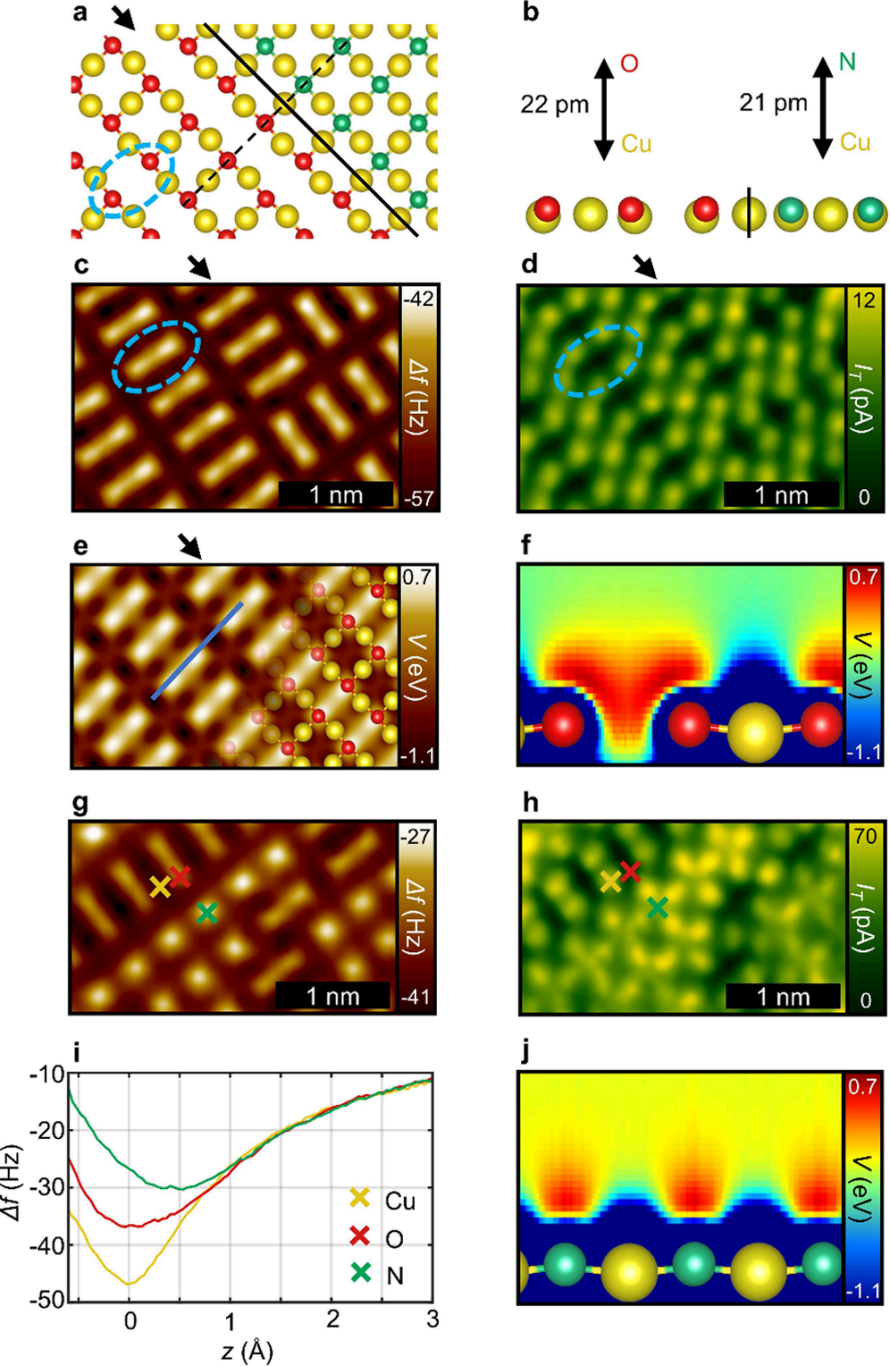
R45°O reconstruction and c(2 × 2)N reconstruction on Cu(100). (a) DFT-optimized structure of the R45°O reconstruction (bottom left) and c(2 × 2)N reconstruction (top right) as top views. For clarity only the top atomic layer is shown. As indicated by the black line, the structures for the copper oxide and the copper nitride systems are obtained from separate DFT calculations. (b) Side view along the dashed line in (a), depicting the relative heights of the surface species. (c) CuOx tip constant height nc-AFM measurement of the ()R45°O reconstruction. (d) Simultaneously recorded tunneling current. (e) Calculated electrostatic potential plotted at a height of 164 pm relative to the topmost surface atom. (f) Cross section of the electrostatic potential along the blue line shown in (e). (g) CuOx tip constant height nc-AFM measurement of the codeposited R45°O reconstruction and c(2 × 2)N reconstruction. (h) Simultaneously recorded tunneling current. (i) Δ*f*(*z*) spectra of the Cu, O, and N sites marked in (g). *z* = 0 corresponds to the tip height of the measurement in (g). (j) Cross section of the electrostatic potential for the c(2 × 2)N reconstruction along the same axis as in (f).

The Cu(100) R45°O surface system again features rows of copper and oxygen atoms, which, however, are separated by alternating added (AR) and missing (MR) rows of copper atoms (Figure 2a lower left and Figure 2b left). Contrary to the previous copper oxide systems, this surface shows two domain orientations with identical atomic structure along the [010] and [001] directions, respectively.^18^ In the nc-AFM measurement in Figure 2c elongated protrusions of the oxygen sites emerge (blue circles in Figure 2), which bridge the missing rows (black arrows in Figure 2), whereas the topographically lower Cu sites appear as weak depressions surrounding the oxygen protrusions. The location of these Cu atoms is further confirmed by an increased tunneling current in the simultaneously recorded STM data (Figure 2d). A relatively small tunneling current is measured overall, caused by the considerably low conductivity of metal oxide surfaces compared with pure metal substrates. The same holds for the CuOx tip, which also exhibits a lower conductivity than a pure metal tip due to the terminating oxygen atom.^18^ Again, the calculated electrostatic potential in Figure 2e shows very good agreement with contrast signatures observed in the nc-AFM measurement (Figure 2c, see also height-dependent measurements in Figure S2), which holds especially for the dimer-like shape of the oxygen atoms. Further examinination of a vertical cross section of the electrostatic potential along the dimer axis (Figure 2f) illustrates how the intermediate missing row distorts the valence electron orbitals of the oxygen atoms, leading to the dimer-like charge density.

As shown by Wofford et al.,^40^ the R45°O structure can coexist with the nitrogen-induced c(2 × 2)N reconstruction^41,42^ on the Cu(100) surface. In the latter case, the nitrogen atoms occupy hollow sites on Cu(100) without the formation of missing or added rows (Figure 2a upper right, Figure 2b right, Supplementary Figure S3). Figure 2g displays an nc-AFM image encompassing both phases, where the dimer-like protrusions of the oxygen atoms coexist with isolated maxima of the nitrogen atoms. Additionally, the simultaneously recorded tunneling current in Figure 2h allows clear allocation of the metal sublattice for both reconstructions (please note the structure of the phase boundary in Figure 2a,b as deduced from these measurements). Corresponding Δ*f*(*z*) spectra are depicted in Figure 2i, which show a clear separation even between the N and O spectra. Again, these findings correlate excellently with the cross sections of the calculated electrostatic potential. In fact, in case of the O dimers, the charge density laterally merges from both sides toward the missing row (Figure 2f), while the charge density above the N sites vertically spreads from the surface (Figure 2j). Importantly, the results discussed so far demonstrate that the nc-AFM contrast with CuOx tips strongly correlates with the local electrostatic potential and the inherent charge density contours, which is the basis for the observed chemically selective contrast.

Contrary to the well-known surfaces discussed above, an approach of the structural elucidation of two coexisting O-induced reconstructions on Ag(111) is given in the Supporting Information. Their atomic structures are largely unknown; thus, they offer an opportunity to explore the applicability of the described chemical selectivity. One measurement addresses the p(4 × 4)O reconstruction, where several contradicting models based on combined STM/DFT data are discussed in the literature.^43,44^ Our CuOx tip nc-AFM measurement shown in Supplementary Figure S4a–d allows developing a model based on AgO_4_ units with different O orientations with respect to the substrate. The second surface structure we investigated shows a striped STM contrast along the Ag(111) high-symmetry axes. While previous studies assumed that such stripes are built from protruding Ag atoms,^44^ our measurement strongly suggests the absence of metal atoms within the top atomic layer (Supplementary Figure S4e–h). Rather, the CuOx-tip nc-AFM contrast features a pore arrangement of protruding oxygen atoms, while the simultaneously recorded tunneling current reveals the underlying Ag sublattice featuring a vacancy structure based on the Ag(111) unit cell.

**Figure 3:**
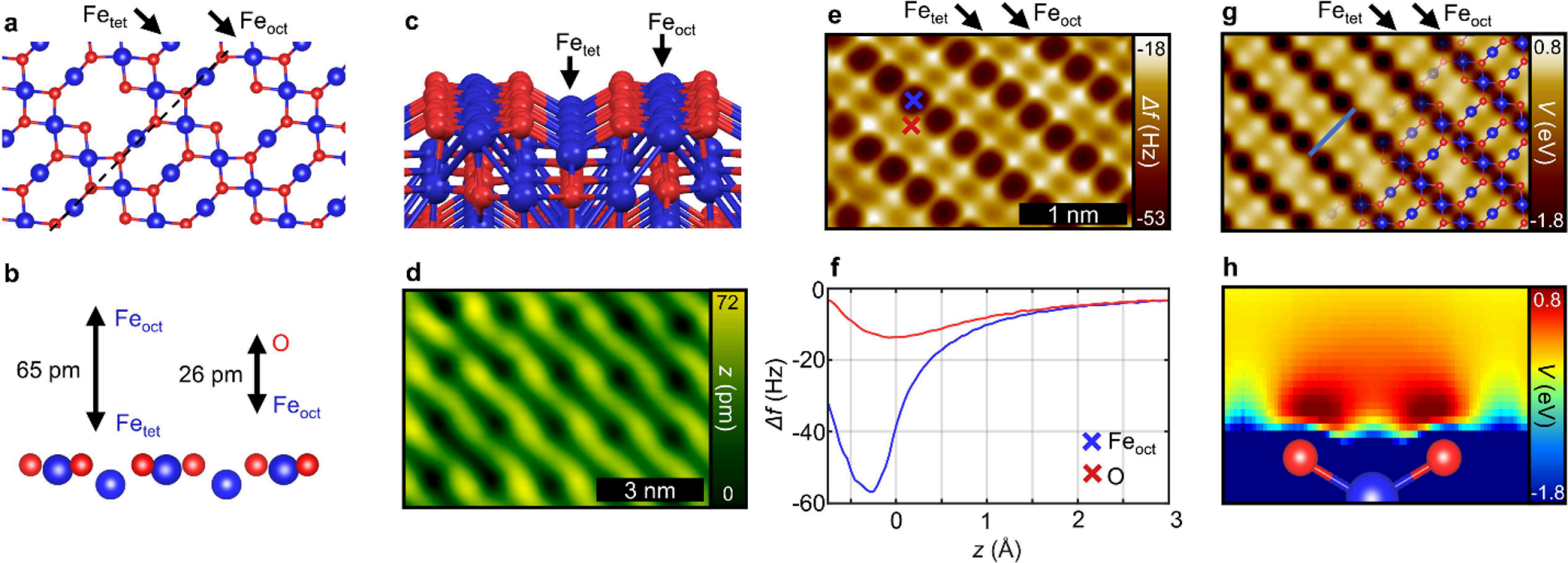
Magnetite Fe_3_O_4_(001). (a) DFT-optimized structure of the subsurface cation vacancy model by Bliem et al.^45^ as a top view. For clarity only the top atomic layer is shown. (b) Side view along the dashed line in (a), depicting the relative heights of the surface species. (c) Perspective of the DFT-optimized structure, which additionally shows the subsurface layer. (d) STM overview image with a feedback loop (1.0 V, 10 pA). (e) CuOx tip constant height nc-AFM. (f) Δ*f*(*z*) spectra of the Fe and O sites marked in (e). *z* = 0 corresponds to the tip height of the measurement in (e). (g) Calculated electrostatic potential plotted at a height of 166 pm relative to the topmost surface atom. (h) Cross section of the electrostatic potential along the blue line in (g).

To generalize our approach to further elements, two different iron oxide surfaces are considered. The magnetite Fe_3_O_4_(001) surface is already well-described by the subsurface cation vacancy model^45^ and holds relevance in various catalytic applications.^13^ The DFT-optimized structure is shown in Figure 3a–c, consisting of alternating rows of octahedral (Fe_oct_) and tetrahedral iron atoms (Fe_tet_). While the Fe_oct_ atoms are almost co planar with the O atoms, the Fe_tet_ atoms are situated 65 pm lower (Figure 3b). In the suggested model, subsurface Fe vacancies reorganize the second layered row of Fe_tet_, resulting in an unoccupied tetrahedral position on every fourth atom. This establishes the R45° periodicity and results in the characteristic distorted STM contrast depicted in Figure 3d. The nc-AFM measurement with a CuOx tip in Figure 3e reveals a distinct contrast between the attractive Fe_oct_ atoms (dark spots) and their surrounding four O atoms imaged as repulsive maxima (bright spots). The Δ*f*(*z*) spectra in Figure 3f feature the typical separation of the curves demonstrating a chemical selectivity over an extended *z* range, very similar to that found for the copper oxide surfaces above. Interestingly, no significant contrast features are observed for the topographically lower Fe_tet_ atoms. Notably, this finding is also reflected in the calculated electrostatic potential (Figure 3g), which again shows excellent agreement with the measurement (Figure 3e, see also height-dependent measurements in Figure S2). An examination of a cross section of the electrostatic potential along the O–Fe_tet_–O axis (Figure 3h) further reveals how the valence electron orbitals and related charge density of the oxygen atoms shield the topographically lower Fe_tet_ atoms toward the surface normal, whereby imaging of these atoms is restricted.

The second iron oxide system that we investigated concerns a surface reconstruction of Fe_3_O_4_(110), where the structure is largely unknown. Under standard preparation conditions Fe_3_O_4_(110) forms [111]-oriented nanofacets, which leads to a strongly corrugated topography in STM experiments.^13^ However, for higher annealing temperatures, this structure can coexist with an atomically flat reconstructed phase.^46,47^ Although constant-height nc-AFM imaging was challenging due to mobile species on this phase, its basic structure could be revealed (Supplementary Figure S5).

**Figure 4:**
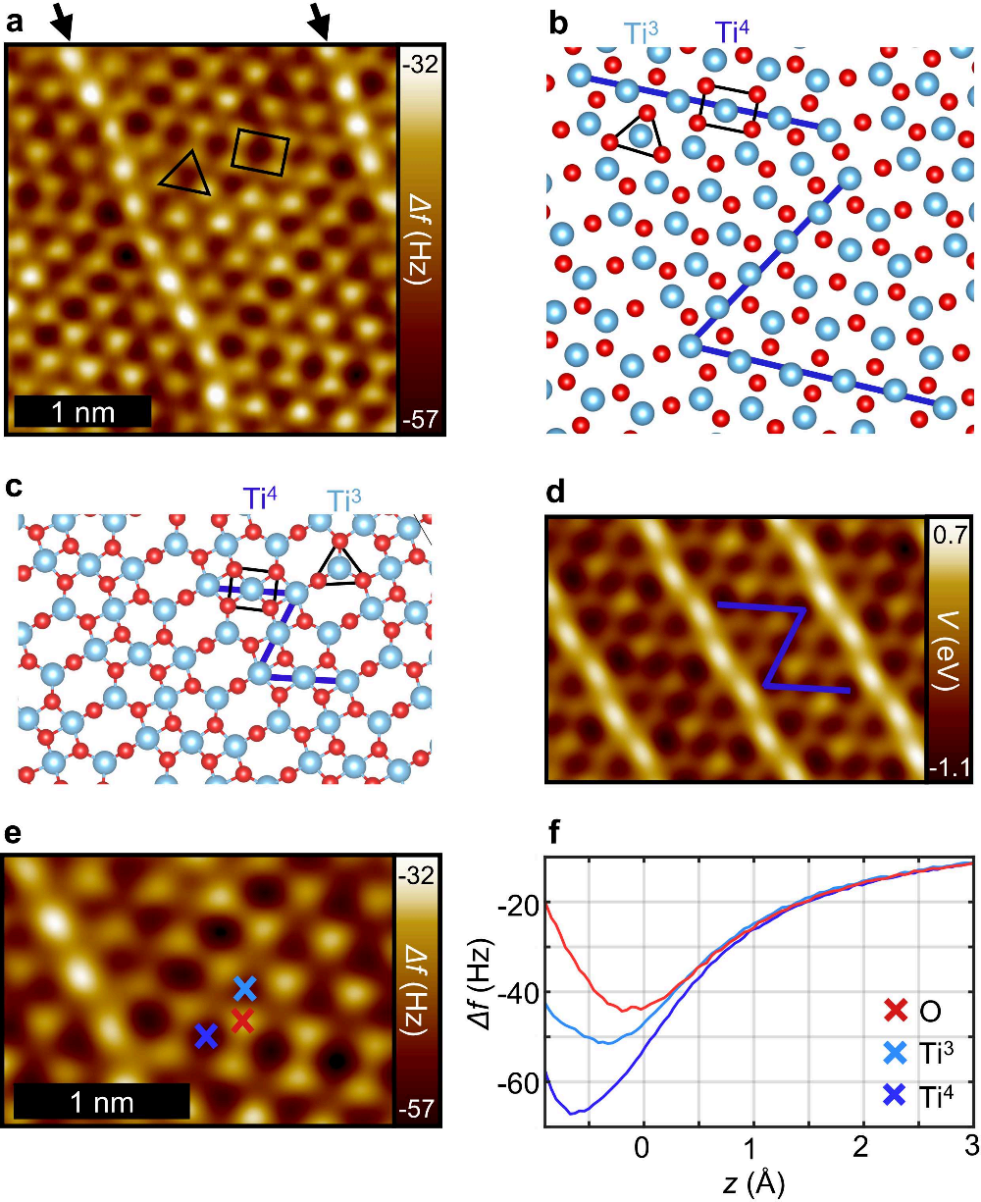
z-TiO_*x*_-phase on Pt(111). (a) CuOx tip constant height nc-AFM of the zigzag structure. (b) Extracted atomic positions from an area of the measurement. The blue line marks the zigzag-like arrangement of the 4-fold coordinated Ti^4^ atoms (square). The 3-fold coordinated Ti^3^ atoms are marked with a triangle. (c) DFT-optimized structure by Barcaro et al.^52^ of a similar structure to the measurement but with a smaller unit cell as top view. For clarity only the top atomic layer is shown. (d) Calculated electrostatic potential of the DFT-optimized structure in (c), plotted at a height of 166 pm relative to the topmost surface atom. (e) Excerpt of CuOx tip constant height nc-AFM. (f) Δ*f*(*z*) spectra of the different surface sites marked in (e). *z* = 0 corresponds to the tip height of the measurement in (a).

To apply our methodology to a surface with a much higher structural complexity compared to the systems considered so far, we also investigated a titanium oxide nanostructure. TiO_*x*_ thin films grown on Pt(111) show various stable surface phases^48−50^ where we focus on one of the so-called zigzag (z) phase. Thereby, the label ”zigzag” refers to a distinct contrast found in STM experiments (see also Supplementary Figure S6). The z phase exists with unit cells of different size and stochiometry where various models are discussed in the literature.^49−52^ An nc-AFM measurement of such a zigzag structure in its maximal complexity is shown in Figure 4a. Based on the results discussed above, the oxygen and titanium sublattices can readily be identified. The atomic positions directly extracted from the measurement are depicted in Figure 4b, showing the complex arrangement of 3-fold- and 4-fold-coordinated titanium atoms (Ti^3^ and Ti^4^, respectively) and 2-fold- and 3-fold-coordinated oxygen atoms. The line segments of the zigzag structure (indicated as blue lines in Figure 4b) possess a length of 6 Ti^4^ atoms. The areas between these lines are filled by differing numbers of Ti^3^ units. Moreover, the periodically repeating zigzag structures are separated by rows of elevated 2-fold-coordinated oxygen atoms as shown by their strongly repulsive contrast and a slight wave-like appearance (black arrows in Figure 4a). This arrangement is in agreement with the DFT calculation by Barcaro et al. of a similar structure but with a smaller unit cell, i.e. with zigzag line segments of a length of 3 Ti^4^ atoms shown in Figure 4c.^52^ Nevertheless, the corresponding electrostatic potential shown in Figure 4d reproduces the most dominant contrast features observed for the more complex structure found in the experiment. Figure 4f shows Δ*f*(*z*) spectra recorded above the sites marked in Figure 4e. It can be observed that the Ti^4^ atoms appear more attractive than the Ti^3^ species. Yet, according to the DFT structure mentioned above, these atoms are assumed to be nearly in the same height range (±3 pm)^52^ (Supplementary Figure S6). Presumably, the more attractive interaction above the Ti^4^ sites is not an effect of topography but is from a distinct charge distribution as a consequence of their higher coordination. Moreover, the minimum of the Δ*f*(*z*) spectrum for the Ti^4^ atom is about 50 pm closer to the surface than for the Ti^3^ atom, despite their nearly identical topography. In this proximity, in addition to electrostatic tip–sample interactions, chemical forces may no longer be negligible.

**Figure 5:**
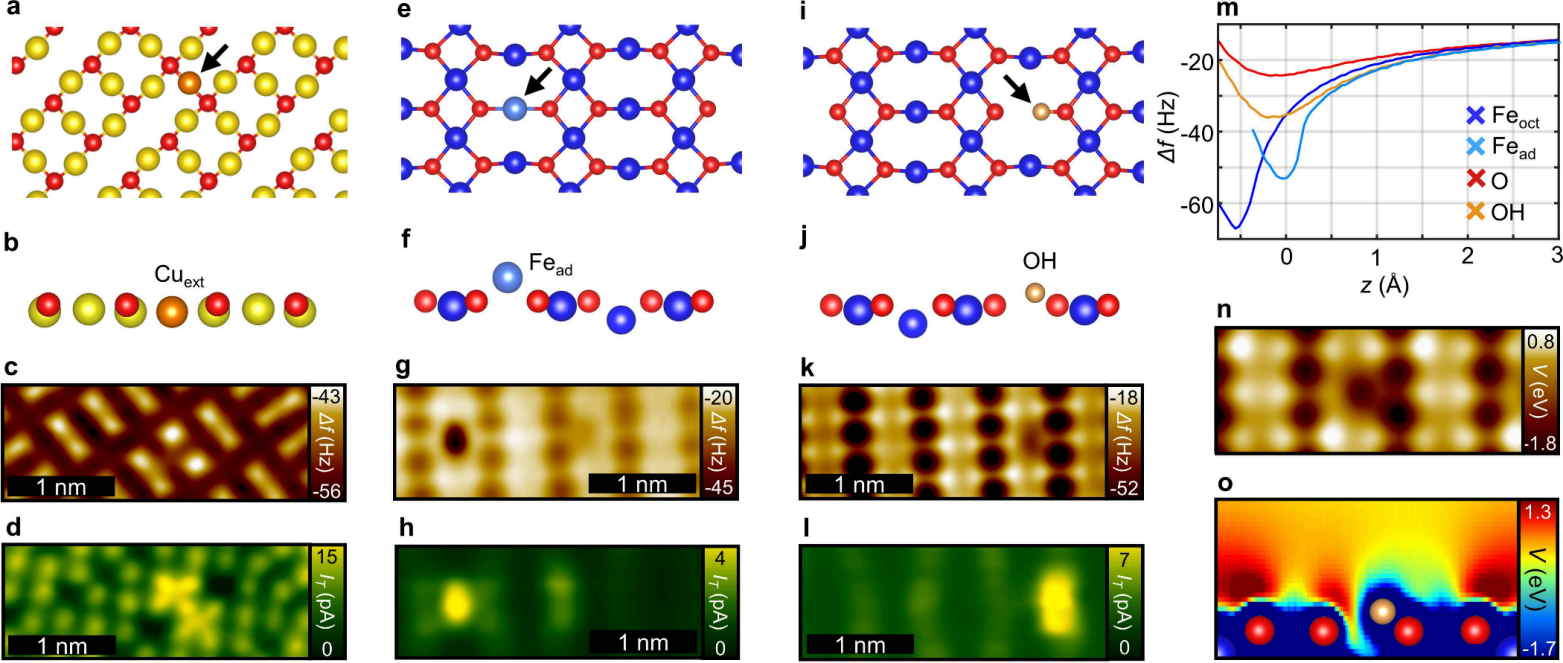
Defect structures. (a) Structural model of the Cu_ext_ defect of the R45°O reconstruction on Cu(100) as a top view. (b) Side view from (a). (c) CuOx tip constant height nc-AFM of the Cu_ext_ defect. (d) Simultaneously recorded tunneling current. (e) Structural model of the Fe_ad_ defect on Fe_3_O_4_(001) as a top view. (f) Side view from (e). (g) CuOx tip constant height nc-AFM of the Fe_ad_ defect. (h) Simultaneously recorded tunneling current (*U* = 0.17 V). (i) DFT-optimized structure of the OH defect on Fe_3_O_4_(001) as the top view. (j) Side view from (i). (k) CuOx tip constant height nc-AFM of the OH defect. (l) Simultaneously recorded tunneling current (*U* = 0.17 V). (m) Δ*f*(*z*) spectra of the Fe, O, and defect sites in (g) and (k). *z* = 0 corresponds to the tip height of the measurement in (g). (n) Calculated electrostatic potential, plotted at a height of 166 pm relative to the topmost oxygen atom. (o) Cross section of the electrostatic potential along the axis of the OH group.

Besides the periodic structures of the metal oxide systems studied so far, the characterization of atomic point defects is of crucial importance for their macroscopic catalytic, optical, and electronic properties.^6,12,13,53−55^ In the following, we exemplify how the chemical selectivity of CuOx tips can be used to characterize defects, which we observed in the systems discussed above. One example can be seen within Figure 5a–-d, which shows a frequently occurring filled-row defect of the R45°O reconstruction (defect free surface in Figure 2). In the case of this well-known defect, a single extra Cu atom (Cu_ext_) fills a missing row site between two oxygen atoms.^56^ The related charge reorganization disrupts the typical dimer-like structure of the neighboring oxygen atoms, leading to two individual protrusions, which appear considerably brighter compared to the dimer-like oxygen pairs in the nc-AFM measurement (Figure 5c). The presence of the extra Cu_ext_ is further confirmed by the simultaneously recorded STM image in Figure 5d, which allows the metal sublattice.

Furthermore, an iron adatom defect (Fe_ad_) can be observed on the Fe_3_O_4_(001) surface (Figure 5e–h, defect free surface in Figure 3). The metallic nature of this common defect^45^ can be identified by the strongly attractive tip–sample interaction, and further confirmed by an increased tunneling current (Figure 5h). In addition, the atomic structure of the underlying Fe_3_O_4_(001) lattice is clearly visible, which allows confirmation that the Fe_ad_ occupies the bulk continuation sites of the Fe_tet_ atoms.^45^

For the defect chemistry of metal oxide materials, hydrogen, which usually forms hydroxyl species, plays an important role.^57^ On the Fe_3_O_4_(001) surface, for instance, hydrogen located at the highly reactive bulk continuation sites of the Fe_tet_ atoms (Figure 5i,j) is the most commonly observed defect on freshly prepared samples.^13^ An nc-AFM image of such a defect is seen in Figure 5k with the corresponding Δ*f*(*z*) spectra in Figure 5m. The formed OH group locally reduces the charge density, leading to a significantly more attractive nc-AFM contrast as compared to the O atoms. The simultaneously recorded STM image in Figure 5l further confirms this defect, as the neighboring iron atoms exhibit a higher tunneling current due to more empty states, as reported in previous studies.^13^ Again, the nc-AFM contrast is excellently reproduced by the calculated electrostatic potential in Figure 5n,o, based on the corresponding DFT-optimized structure.

## Conclusions

By investigating a variety of different metal oxide surfaces with increasing structural complexity, we demonstrate that nc-AFM imaging with CuOx-functionalized tips allows us to directly reveal the metal and oxygen sublattices. Even for systems with atoms at various relative heights as well as surfaces with a high degree of structural complexity or atomic-scale defects, we obtain a direct assignment of the atomic configurations. The excellent agreement between the nc-AFM data and the calculated electrostatic potential allows explaining the contrast mechanism by the negative charge at the O-terminated tip apex^36^ and its interaction with the charge density of the surfaces. The successful application of the methodology to defects and surface systems where so far no conclusive models exist demonstrates the universal applicability of the experimental approach. Our results not only establish CuOx tips for the chemically selective characterization of metal oxide surfaces but also contribute to the standardization of chemical surface imaging techniques on the atomic scale.

## Methods

A low-temperature scanning probe microscope (SPM) with the MATRIX SPM control system (LT-STM/AFM) from Scienta Omicron was used for the experiments. The microscope was operated under ultrahigh vacuum conditions with a base pressure below 5 × 10^–11^ mbar and cooled to 78 K by a liquid nitrogen bath cryostat. The qPlus force sensors^30^ used enabled simultaneous STM and AFM data recording with resonance frequencies *f*_0_ of 26–28 kHz and quality factors of 5k–15k. Unless otherwise stated, the AFM experiments were performed with a constant amplitude (1.0 Å) active feedback loop and in constant height mode with a bias voltage of 0 V. An STM feedback loop was used for an overview of STM images and to find a suitable position before starting a constant height nc-AFM measurement. To prevent tip changes, low tunneling current set points of 10 pA and bias voltages of 1–2 V were used. Raw image data were processed with a Gaussian filter (Scanning Probe Image Processor, SPIP5.1).

To form a CuOx tip, chemically and focused ion beam (FIB) etched tungsten tips with a tip diameter of less than 30 nm were used. By voltage pulses up to 6 V and indentations up to 2 nm on a Au(111) surface, a clean metallic tip apex is shaped. Afterward, the tip-forming process was continued on the partially oxidized Cu(110) (2 × 1)O surface (for preparation see below). With a set point of 100 mV and 50 pA in STM feedback, a copper oxide cluster is picked up by voltage pulses up to 4 V and indentations up to 4 nm into the oxide stripes. By further indentations within a range of 0.2–0.6 nm, the copper oxide cluster is then formed into a CuOx tip. Before recording all of the data shown, a constant height nc-AFM measurement of an oxide stripe on the Cu(110) (2 × 1)O surface according to Figure 1c is taken to confirm the covalent tetrahedral bonding configuration of the terminal oxygen and its typical imaging contrast.^22,36,58^ This makes the ex situ CuOx tip-forming procedure highly reproducible, and the correct termination can always be ensured, independent from the analyzed sample system. Furthermore, the fingerprint can also be used to determine tip asymmetries, which can then be corrected by further slight indentations. If the oxygen coverage of the Cu(110) sample is greater than 30%, the O/Cu ratio at the apex may rise during tip forming, resulting in tip asymmetries occurring more frequently. The tip must then be reset to a sharp metal tip using the cleaning procedure on Au(111) described above.

For the sample preparation, the Cu(110), Cu(100), Fe_3_O_4_(001), and Pt(111) single crystals were cleaned by cycles of Ar^+^ sputtering and subsequent annealing up to 600 K, each for 10 min, before the oxidation processes were performed. For the specific surface reconstructions, a detailed instruction is given below.

### Cu(110)-Reconstructions

After the final annealing step of the cleaning procedure, the sample temperature was lowered to 450 K. At this temperature molecular O_2_ was dosed with a pressure of 2 × 10^–8^ mbar for 10–15 s to induce the (2 × 1)O reconstructed oxide stripes. For the (6 × 2)O reconstruction, which requires further oxidation, the cleaned Cu(110) crystal was heated to 500 K, before molecular O_2_ was dosed with a pressure of 5 × 10^–6^ mbar for 10 min.

### Cu(100)-Reconstructions

After cleaning, the Cu(100) crystal was heated to 400 K and molecular O_2_ was dosed with a pressure of 1 × 10^–6^ mbar for 20 min to achieve the R45°O reconstruction. For the c(2 × 2)N reconstruction, the cleaned Cu(100) crystal was sputtered with nitrogen at 500 eV for 30 s, followed by annealing at 500 K for 10 min. For the codeposited surface measured in Figure 2g, subsequently, molecular O_2_ was dosed with a pressure of 1 × 10^–6^ mbar for 5 min at an annealing temperature of 400 K. It should be mentioned here that the chemisorption of nitrogen on Cu(100) can only be achieved by sputtering highly energetic nitrogen ions on the surface and does not occur spontaneously from the gas phase as it does for oxygen. Furthermore, for the sample preparation, two different sputter guns for argon and nitrogen sputtering with separated gas lines were used. Therefore, a nitrogen contamination within the measurements of the pure R45°O reconstruction including the Cu_ext_ defect can be excluded.

### Fe_3_O_4_(001)

Ar^+^ sputtering and UHV-annealing might reduce the O/Fe ratio, which hinders the surface in reconstruction. To prevent this, for the last cleaning cycle, the annealing temperature was increased to 900 K for 30 min. In the first 20 min of annealing, molecular oxygen was dosed with 5 × 10^–7^ mbar, while for the last 10 min, UHV annealing was performed.

### TiO_*x*_ Thin Films

Titanium was deposited on a clean Pt(111) single crystal by electron-beam evaporation of a Ti rod at a deposition rate of 1 ML per minute for 40–50 s. Afterward O_2_ was dosed at a sample temperature of 900 K and a pressure of 1 × 10^–5^ mbar for 10 min to form TiO_*x*_ structures on the surface. For the measured zigzag phase in Figure 4, the surface was reduced by UHV annealing at 1050 K for 10 min.

### Computational Details

Utilizing the Vienna Ab Initio Simulation Package (VASP) for periodic density functional theory (DFT) calculations,^59,60^ atomistic investigations were conducted on the metal oxide surfaces focusing on the local potential *V*(*r*) calculated as
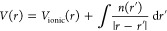
with the electron density *n*(*r*), the ionic potential *V*_ionic_(*r*), and the Hartree potential (second term) of the surfaces. The tip and associated tip–sample interactions such as dipole/multipole effects, chemical forces, and Pauli repulsion are not included. In all calculations, the Perdew–Burke–Ernzerhof (PBE) GGA function,^61^ together with projected augmented wave (PAW) pseudopotentials and a plane-wave cutoff of 600 eV for the wave functions, was employed. A Fermi smearing with a standard deviation of 0.2 eV described the electronic states. Dispersion effects were taken into account by using the DFT-D3 method with Becke–Johnson damping function.^62^ Monkhorst–Pack k-point sampling with an 8 × 8 × 1 mesh was employed for Brillouin zone integration in all calculations. For the copper-based systems, including the CuOx tip, the geometries were fully optimized (keeping only the bottom layers at a bulk lattice constant of 3.64 Å). Except for the OH defect on Fe_3_O_4_(001), which was optimized, the geometry for the Fe_3_O_4_(001) and TiO_*x*_ surfaces were taken from Bliem et al.^45^ and Barcaro et al.,^52^ respectively. For all calculations, a dipole correction to the energy for the slab model was carried out perpendicular to the surface. The convergence criteria for the forces on the nuclei were 0.01 eV Å^–1^ for geometry optimization and 10^–8^ eV for electronic relaxation in all calculations.

## Supporting Information Available

Height-dependent AFM of the (2 × 1)O reconstruction on Cu(110), R45°O reconstruction on Cu(100) and the magnetite Fe_3_O_4_(001) surface, AFM/DFT of c(2 × 2)N reconstruction on Cu(100), STM/AFM of Ag(111) oxide phases, STM/AFM of Fe_3_O_4_(110), and STM/DFT of z-TiO_*x*_ on Pt(111) (PDF)
