# Supplementary material for: Standardization of Chemically Selective Atomic Force Microscopy for Metal Oxide Surfaces
Source: ACS Nano. 2024 Aug 5;18(33):21948–56. doi: 10.1021/acsnano.4c03155 (PMC11342932; doi:10.1021/acsnano.4c03155)
Supplement: Supplementary file 1 — nn4c03155_si_001.pdf [file nn4c03155_si_001.pdf]

Supporting Information

Standardization of chemically selective atomic force microscopy for metal-oxide surfaces

Philipp Wiesener,[†,‡] Stefan Förster,[¶] Milena Merkel,[†,‡] Bertram Schulze Lammers,[†,‡] Harald Fuchs,[†,‡] Saeed Amirjalayer,[§,‖] and Harry Mönig[*,†,‡]

†*Universität Münster, Physikalisches Institut, Münster 48149, Germany*

‡*Center for Nanotechnology, Münster 48149, Germany*

¶*Martin-Luther-Universität Halle-Wittenberg, Institut für Physik, Halle 06120, Germany*

§*Universität Münster, Institut für Festkörpertheorie, Münster 48149, Germany*

‖*Center for Multiscale Theory and Computation, Münster 48149, Germany*

E-mail: *harry.moenig@uni-muenster.de

Contrast dependence on the tip-sample distance

An important aspect for understanding the strong correlation between the electrostatic potential and the chemically selective nc-AFM contrast is the tip-sample distance. As a rough orientation, we found that imaging at a tip-height around the minimum of the $\Delta f(z)$-curves (*i.e.* the inflection point of the force-distance curve $F(z)$) above the metal atoms results in the most distinct contrast for metal- and oxygen atoms. Considering the overlap of the charge contours of the valence electrons of both, the surface and the tip (Fig. 1f) as well as DFT simulated force spectra[1] we estimated the tip-sample distances to be within a range of 320 pm ± 20 pm (see main text). To further elucidate the height range within which

chemically selective contrast can be expected Fig. S1 shows height dependent nc-AFM data from the (2x1)O-reconstruction on Cu(110) allowing a direct comparison with the corresponding electrostatic potential. Fig. S1a shows a map of $\Delta f(z)$ data along an added row ([001]-direction), which are background corrected using a $\Delta f(z)$-spectrum recorded on the bare Cu(110) plane. Over a decent height range, the electrostatic potential shown on the right of Fig. S1a is well reproduced. By approaching further to the surface, repulsive and chemical interactions start to dominate, which lowers the contrast between the metal- and oxygen surface species.[1] Figure S1b shows a comparison of constant-height nc-AFM data with corresponding maps of the electrostatic potential showing good agreement for the identical relative heights.

Furthermore, in Figure S2, nc-AFM constant height measurements for the $(2\sqrt{2}\,\mathrm{x}\,\sqrt{2})\mathrm{R}45°$O-reconstruction on Cu(100) and the magnetite Fe_3O_4(001) surface at different heights are shown, which correspond well with the height-evolution of the electrostatic potential. Especially for large tip-sample distances (Fig S2a first row, Fig S2b) the agreement with the electrostatic potential is good, while approaching significantly further to the surface than the $\Delta f(z)$-minimum of the metal species, emerging repulsive forces lead to a different imaging contrast and a mismatch with the electrostatic potential (Fig. S2a last row).

Ag(111)-oxide phases

Another metal-oxide system we investigated is the p(4x4)O-reconstruction on Ag(111) where several contradicting models based on combined STM/DFT data exist.[2,3] The most recent work by Andryushechkin et al.[2] for example proposes a structure based on Ag_6O and Ag_3O_x units to explain the observed STM contrast, which shows a pronounced bias dependence. For the preparation, a significantly higher oxygen pressure (1 x 10^{-2} mbar O_2 for 20 min at 500 K) is required compared to the systems shown in the main paper. STM overview images of this phase are depicted in Fig. S4a and b, while a constant-height nc-AFM

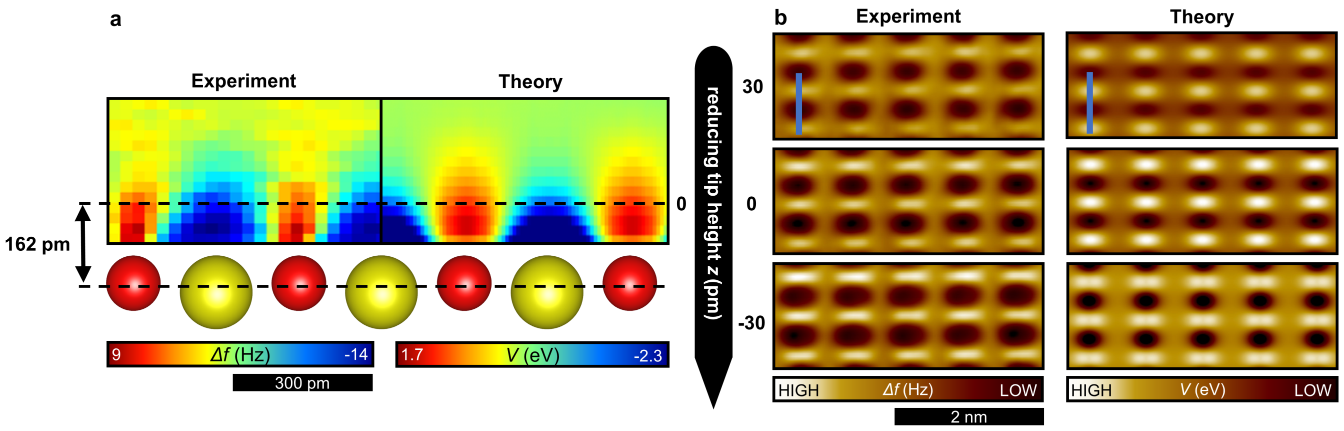

Figure S 1: **Tip-sample distance dependence on the chemical selective contrast. a** Left: Map of experimental CuOx-tip $\Delta f(z)$ data from the (2x1)O-reconstruction on Cu(110) along an added row ([001]-direction); background corrected using a $\Delta f(z)$-spectrum recorded on the bare copper. Right: Cross section of the corresponding electrostatic potential. Atomic positions are given below: copper (yellow) and oxygen (red) atoms. $z = 0$ corresponds to the tip height of the measurement in Fig. 1c, roughly around the $\Delta f(z)$-minimum of the metal species. **b** CuOx-tip constant height nc-AFM for different tip heights (left) and corresponding electrostatic potential (right). The blue lines mark the cross section shown in a.

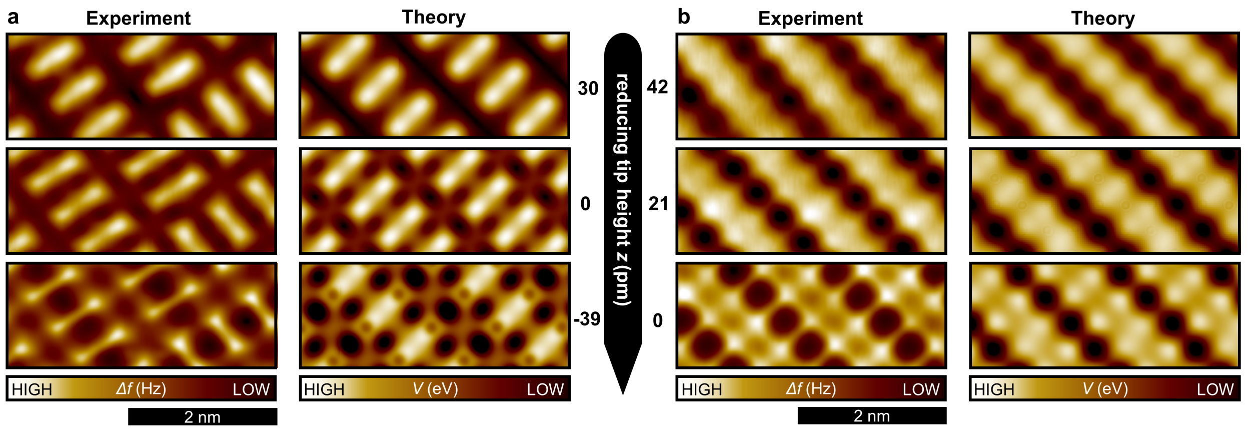

Figure S 2: **Height depended nc-AFM images.** CuOx-tip constant height nc-AFM for different tip heights (left) and corresponding electrostatic potential (right) for **a** the $(2\sqrt{2}$ x $\sqrt{2})$R45°O-reconstruction on Cu(100) and **b** the magnetite $Fe_3O_4(001)$ surface. $z = 0$ corresponds to the tip height of the measurements in the main paper, roughly around the $\Delta f(z)$-minimum of the metal species.

measurement conducted with a CuOx-tip is displayed in Fig. S4c. The nc-AFM data reveal the presence of elevated Ag atoms, which can be identified by the typical strongly attractive tip-sample interaction (dark spots). These dark spots are surrounded by four weak protrusions, which suggests that the basis of this reconstruction consists of AgO_4 units

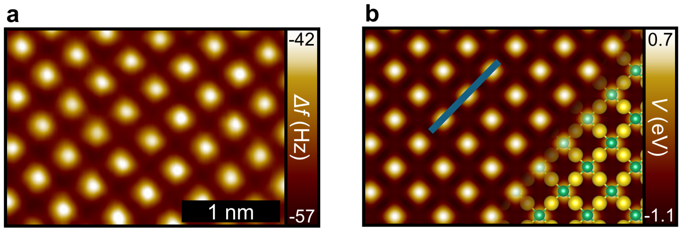

Figure S 3: **c(2x2)N-reconstruction on Cu(100).** **a** CuOx-tip constant height nc-AFM of the c(2x2)N-reconstruction. **b** Calculated electrostatic potential (plotted at a height of 164 pm relative to the top most nitrogen atom) and DFT structure (bottom right) of the c(2x2)N-reconstruction. The blue line marks the cross section shown in Fig. 2j.

with different O-orientations with respect to the Ag(111) lattice. A schematic model of this atomic arrangement as derived from the nc-AFM measurement is shown in Fig. S4d.

Coexisting with the p(4x4)-phase, another silver-oxide reconstruction is observed. The overview STM image shown in Fig. S4e reveals a linear phase modulated with dark stripes. Schnadt et al. associate this striped phase with elevated silver atoms.[3] Contrary, our CuOx-tip nc-AFM data do not show distinct contrast features, which indicate the presence of any metal atoms within the top atomic layer. Yet, the nc-AFM measurement shown in Fig. S4f reveals a surface structure which is purely terminated by hexagonal arranged protruding oxygen atoms. In addition, the simultaneously recorded tunneling current in Fig. S4g shows Ag-vacancies in the underlying Ag(111)-lattice. By that, the structural model in Fig. S4h can be derived, where the oxygen atoms are located on the hollow sites of the Ag(111) (1x1)-lattice. Please note the slight variations in the brightness of the oxygen atoms (Fig. S4f), which indicate a modulation in height. This modulation is probably the origin for the striped contrast found in the STM data and could be a consequence of strain effects within the top atomic layers.

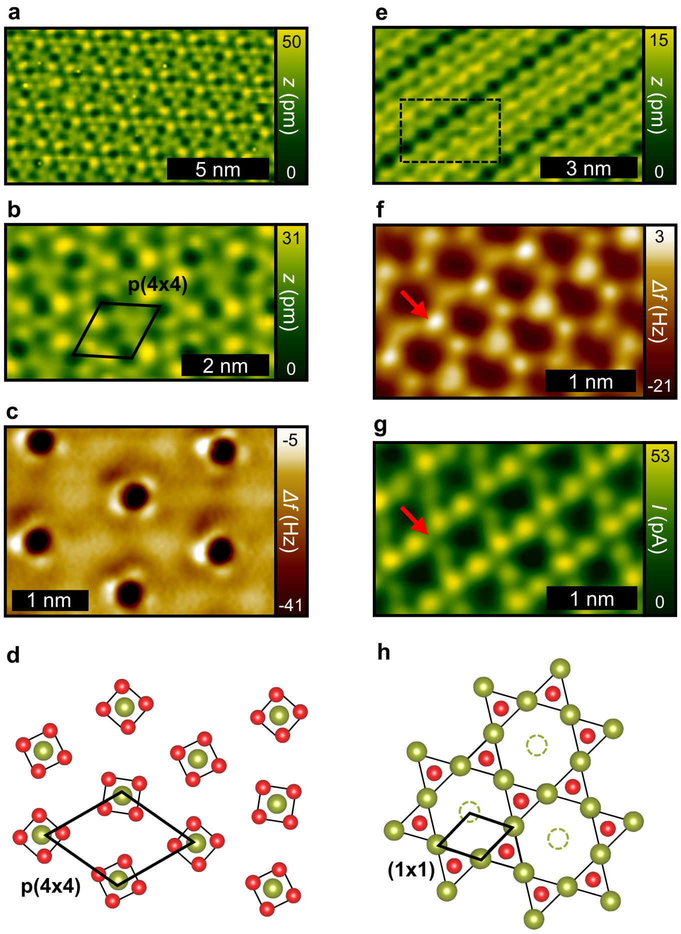

Figure S 4: **Ag(111)-oxide phases.** **a** STM overview image with feedback loop (1.0 V, 50 pA) of the p(4x4)O-reconstruction on Ag(111). **b** Small scale STM image showing the p(4x4) periodicity. **c** CuOx-tip constant height nc-AFM measurement of the p(4x4)-reconstruction. **d** Structural model of the p(4x4)-reconstruction derived from the nc-AFM measurement. **e** STM overview image with feedback loop (2.0 V, 10 pA) of a stripe phase on Ag(111). The dashed rectangle marks the constant height measurement. **f** CuOx-tip constant height nc-AFM measurement of the stripe phase. **g** Simultaneously recorded tunneling current. The red arrow indicates the position of an oxygen atom. **h** Structural model of the stripe phase derived from the nc-AFM and STM measurement. Ag vacancies in the pores are indicated by dashed circles.

$Fe_3O_4(110)$

The magnetite $Fe_3O_4(110)$ surface, usually features the well-known (1x3)-reconstruction and related (111) nanofacets.[4–6] However, as noted by two different studies,[5,6] also a less corrugated structure forms for higher annealing temperatures (here we used 1200 K), coexisting with the faceted (1x3)-reconstruction. The overview STM image in Fig. S5a shows both phases, where the distance between the strongly corrugated stripes of the (1x3)-reconstruction (X-phase) agrees with the one between the nano-facets observed in literature.[4–6] The same holds for the apparent height difference of about 3 Å between (1x3)-reconstructed areas and the less corrugated structure (Y-phase).[6] A high-resolution STM image recorded on one of the flat areas (Y-phase) is shown in Fig. S5b featuring parallel strands along the [-110]-direction with regularly arranged pores. Due to mobile species on this surface, predominantly physisorbed within these pores (see also Fig. S5c), imaging in constant-height mode was challenging. Nevertheless, a small area (blue rectangle in Fig. S5b and c) could be imaged with a CuOx-tip by nc-AFM (Fig. S5d), revealing that the strands consist of threefold coordinated iron atoms. A structural model derived from the nc-AFM measurement is shown in Fig. S5e.

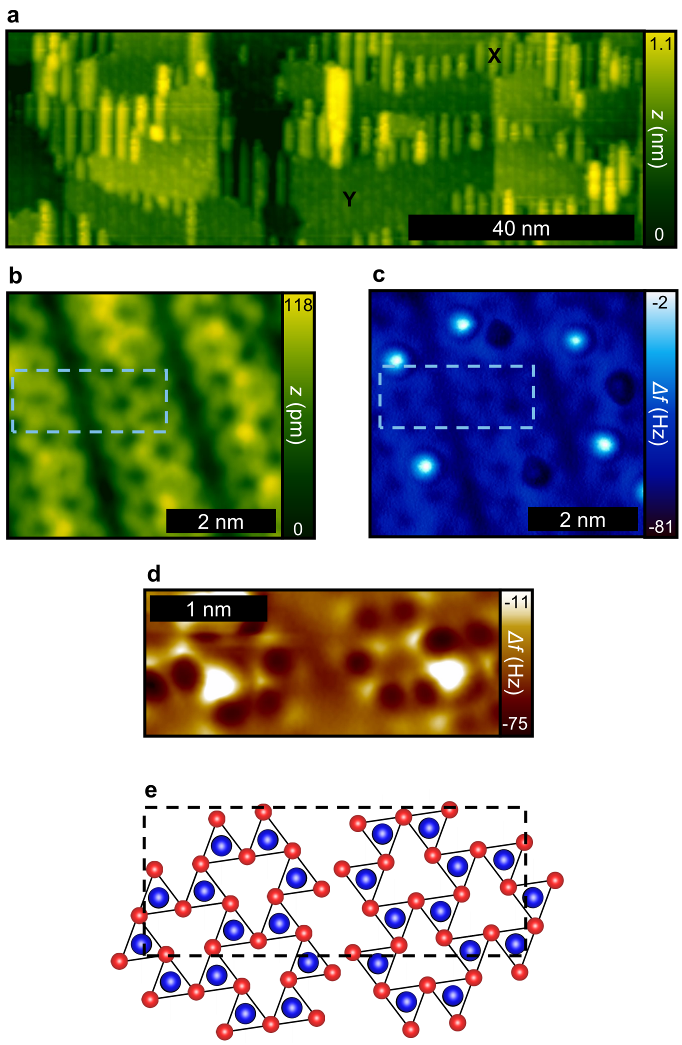

Figure S 5: **Magnetite Fe_3O_4(110).** **a** STM overview image with feedback loop (1.5 V, 10 pA) of the Fe_3O_4(110) surface. Two different structures (X-phase and Y-phase) can be observed. **b** Small scale STM image of the Y-phase. **c** Simultaneously recorded nc-AFM with STM feedback loop. The measurement allows imaging the surface species within the pores, which show tip-induced mobility in the constant-height nc-AFM measurement and appear invisible in constant-current STM. **d** CuOx-tip constant height nc-AFM measurement of the area in the dashed line in b and c. **e** Structural model derived from the nc-AFM measurement of one of the strands shown in d.

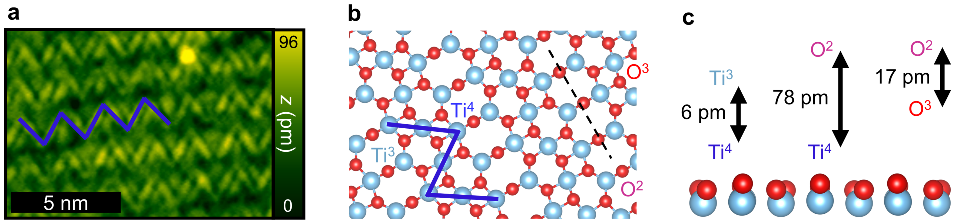

Figure S 6: **z-TiO_x on Pt(111).** **a** STM overview image with feedback loop (2.0 V, 10 pA) showing the zigzag pattern. **b** DFT optimized model by Barcaro et al.[7] of a similar structure to the measurement in Fig. 4 but with a smaller unit cell, i.e. with zigzag line segments with a length of 3 foufold-coordinated Ti^4 atoms as top view. Furthermore the threefold coordinated Ti^3 atoms are labeled as well as the threefold coordinated O^3 atoms and the twofold coordinated O^2 atoms. **c** Side view along the dashed line in b, depicting the relative heights of the surface species.

References

(1) Schulze Lammers, B., Yesilpinar, D.,Timmer, A., Hu, Z., Ji, W., Amirjalayer, S., Fuchs, H., and Mönig, H. Benchmarking atomically defined AFM tips for chemical-selective imaging. *Nanoscale* **2021**, *13*, 13617–13623.

(2) Andryushechkin, B. V., Pavlova, T. V., and Shevlyuga, V. M. New insights into the structure of the Ag(111)-p(4 × 4)-O phase: high-resolution STM and DFT study. *Phys. Chem. Chem. Phys.* **2024**, *26*, 1322–1327.

(3) Schnadt, J., Knudsen, J., Hu, X. L., Michaelides, A., Vang, R. T., Reuter, K., Li, Z., Lægsgaard, E., Scheffler, M., and Besenbacher, F. Experimental and theoretical study of oxygen adsorption structures on Ag(111). *Phys. Rev. B* **2009**, *80*, 75424.

(4) Parkinson, G. S., Lackner, P., Gamba, O., Maaß, S., Gerhold, S., Riva, M., Bliem, R., Diebold, U., and Schmid, M. Fe_3O_4(110)–(1×3) revisited: Periodic (111) nanofacets. *Surf. Sci.* **2016**, *649*, L120–L123.

(5) Jansen, R., Brabers, V.A.M., and van Kempen, H. One-dimensional reconstruction observed on Fe_3O_4(110) by scanning tunneling microscopy. *Surf. Sci.* **1995**, *328*, 237–247.

(6) Walls, B., Lübben, O., Palotas, K., Fleischer, K., Walshe, K. and Shvets, I. V. Oxygen vacancy induced surface stabilization: (110) terminated magnetite. *Phys. Rev. B* **2016**, *94*, 165424.

(7) Barcaro, Gi., Sedona, F., Fortunelli, A., and Granozzi, G. Structure of a TiO_x zigzag-Like monolayer on Pt(111). *J. Phys. Chem. C* **2007**, *111*, 6095–6102.
